# Novel Compound-Forming Technology Using Bioprinting and Electrospinning for Patterning a 3D Scaffold Construct with Multiscale Channels

**DOI:** 10.3390/mi7120238

**Published:** 2016-12-21

**Authors:** Yuanshao Sun, Yuanyuan Liu, Shuai Li, Change Liu, Qingxi Hu

**Affiliations:** 1 Rapid Manufacturing Engineering Center, Shanghai University, Shanghai 200444, China; yj_linda@163.com (Y.S.); allenle1991@shu.edu.cn (S.L.); change_liu2@163.com (C.L.); 2 Shanghai Key Laboratory of Intelligent Manufacturing and Roboties, Shanghai University, Shanghai 200444, China

**Keywords:** vascularization, tissue engineering, multiscale channels, 3D bioprinting, human umbilical vein endothelial cells (HUVECs)

## Abstract

One of the biggest challenges for tissue engineering is to efficiently provide oxygen and nutrients to cells on a three-dimensional (3D) engineered scaffold structure. Thus, achieving sufficient vascularization of the structure is a critical problem in tissue engineering. This facilitates the need to develop novel methods to enhance vascularization. Use of patterned hydrogel structures with multiscale channels can be used to achieve the required vascularization. Patterned structures need to be biocompatible and biodegradable. In this study, gelatin was used as the main part of a hydrogel to prepare a biological structure with 3D multiscale channels using bioprinting combined with selection of suitable materials and electrostatic spinning. Human umbilical vein endothelial cells (HUVECs) were then used to confirm efficacy of the structure, inferred from cell viability on different engineered construct designs. HUVECs were seeded on the surface of channels and cultured in vitro. HUVECs showed high viability and diffusion within the construct. This method can be used as a practical platform for the fabrication of engineered construct for vascularization.

## 1. Introduction

A major focus in the field of tissue engineering is to build new functional tissues with the aim to repair missing or damaged tissues using three-dimensional (3D) cell culture systems, which are designed to mimic the in vivo cell environment [1]. The vascular network supplies nutrition, oxygen, and various factors to complicated, thick tissues and organs [2]. Construction of complex tissues, including that of a vascular network, is a core issue in the field of tissue engineering and regenerative medicine [3]. Fabrication of hollow channels within hydrogel is one such promising strategy to facilitate vascularization. Enhanced vascularization of an in vitro-generated construct has been achieved by culturing endothelial cells embedded in these preexisting channels perfused with nutrients [4,5]. One approach to enhancement in vascularization can be achieved by fabrication of hollow fibers to repair the damaged vessel directly. For example, this can be achieved by using a coaxial flow of two materials such as alginate and calcium chloride solution. In this method, alginate fibers are fabricated using a microporous membrane, which allows ease in control of the fiber diameter during fabrication. The use of solid freedom form has also been reported for patterning 3D structures, using these hollow fibers for studying the 3D vascularization problem. Rapid prototyping based on inkjet printing has been reported as a method for fabricating hollow fibers [6,7,8,9]. In recent years, another method, which relies on patterning the channel network within a hydrogel matrix, has been proposed. Here, sacrificial materials, such as gelatin and Pluronic F127, were used to pattern channels within the hydrogel. Temperature control was used to eliminate the sacrificial material and pattern the network within the hydrogel. Using this method, a single channel size was maintained in the 3D matrix [10,11].

In this study, a bioprinter was used to control patterning of the hydrogel and hollow channel construction (i.e., to print the vascularization structure with 3D hollow channels), which was combined with electrostatic spinning to pattern microchannels. This matrix was seeded with human umbilical vein endothelial cells (HUVECs). Connectivity of channels, degradation rate, and viability of seeded cells were tested for the printed 3D structure.

### Background

Extensive research has been reported in literature related to the preparation of scaffolds for vascularization. Based on this research, bioprinting stands out as a key method used for the preparation of scaffolds. Bioprinting is defined as a computer-aided process for layer-by-layer patterning and assembling of living and nonliving materials [12]. Bioprinting technology resolves limitations that exist for current methods used in the field of tissue engineering. Existing technology requires preparation of a biological or biomimetic structure, however, their integration for organ engineering has proven to be difficult. A method to achieve sufficient vascularization is a critical and challenging problem that needs to be overcome in the pursuit of engineering an organ [13]. Current promising technologies in bioprinting utilize laser, inkjet, or extrusion/deposition techniques. The inkjet-based bioprinting technique is restricted by the upper limit of the bioink viscosity, which is low [14]. A major limitation of the laser-based bioprinting technique is related to the heat generated from laser energy, which may damage cells or affect cell activity. Laser-based bioprinting techniques have limited printing capability in the third dimension. This is a result of the long fabrication time due to gravitational and random setting of cells in the precursor solution [15]. Bioprinting based on extrusion is an ideal technique, which has been successfully used for last 10 years [16]. Extrusion-based bioprinting has been used to dispense various types and viscosities of bioinks by simply adjusting pressure and valve gating time. Moreover, the electrospinning technique has been used as an efficient processing method to manufacture nanoscale or submicron fibrous structures, which serve as the sacrificial material in the extracellular matrix (ECM). This allows improvement in the cellular infiltration toward the center of the scaffold [17,18].

It is important to note that biomaterial development is a critical aspect of bioprinting. The biomaterial used needs to fulfill the biological requirements necessary for cells, along with having favorable mechanical properties and printability [19]. In previous studies, both hydrogel and hydrogel-free cell aggregates have been used for bioprinting. Hydrogel has been demonstrated as a preferred material which fulfills the biomaterial requirements. In addition to this, hydrogel has been widely used in tissue engineering since it is natural, biodegradable, and biocompatible [20].

## 2. Materials and Methods

### 2.1. Materials

Gelatin (gel, from bovine skin, Type B) was obtained from Ruitaibio Co. (Beijing, China), and Pluronic F127 was obtained from Sigma-Aldrich, Inc. (St. Louis, MO, USA); polycaprolactone (PCL), dichloromethane (DCM), and *N*,*N*-dimethylformamide (DMF) were purchased from Sinopharm Chemical Reagent Co. (Shanghai, China). Microbial transglutaminase (mTG, Activa-TI, Ajinomoto Inc., Shanghai, China) was prepared as the crosslinking agent.

### 2.2. Cell Preparation and Culture

HUVECs were obtained from Sinopharm Chemical Reagent Co. (Shanghai, China). These cells were cultured in Roswell Park Memorial Institute (RPMI) 1640 medium supplemented with 10% (*v*/*v*) fetal bovine serum, 100 U/mL of penicillin, and 100 μg/mL streptomycin (Invitrogen, Carlsbad, CA, USA) at 37 °C with 5% (*v*/*v*) CO_2_ in a humid atmosphere. Culture media in a Φ100 mm culture dish (Falcon, New York, NY, USA) was replaced every 3 days. Cells were detached from cell culture dishes using 0.25% trypsin–EDTA (Life Technologies, New York, NY, USA) and cells were collected by using a centrifuge at 1000 rpm.

### 2.3. Bioprinting Setup

**Figure 1:**
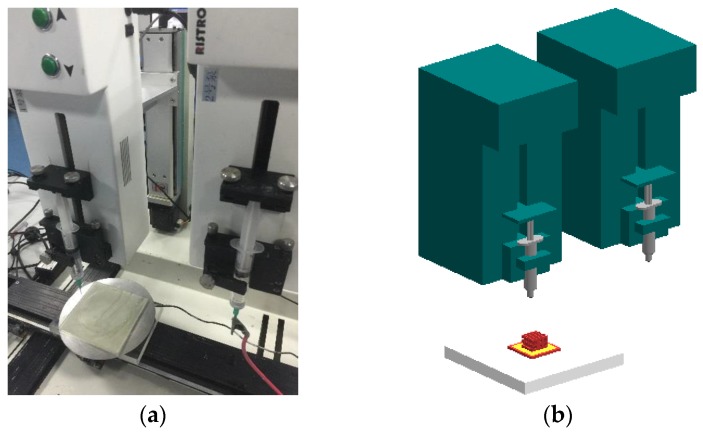
Bioprinting system: (**a**) image of actual system; (**b**) 3D schematic of the syringes.

The bioprinting system is described and shown in Figure 1. The bioprinting system was composed of separate bioprinting and control areas. The 3D bioprinting machine has a five-axis motion-control stage and two micropumps (Figure 1a). Figure 1b shows a 3D schematic of the system. The system used two needle-removable syringes as the ejection nozzle. A needle size of 25 gauge was used. The print nozzle was mounted on a motorized linear Z-stage, whereas the receiving platform was mounted on a separate motorized linear Z-stage. Nozzle tip position along the *x–y* axis, nozzle movement, and ejection velocity were controlled to pattern the structure.

Gelatin was used as a main structure for printing. Gelatin powder was dissolved in distilled water (18 wt %). At this concentration, gelatin reversibly solidifies at room temperature and liquefies at 40 °C. Before printing, mTG was added to the solution such that the final weight ratio of mTG and gelatin in the mixed solution was 1:10. Pluronic F127 was used as a sacrificial material to create fluidic channels. Pluronic F127 powder was dissolved in ice distilled water by continuous stirring for 30 min. The final concentration of the Pluronic F127 was kept to 35 wt %. Pluronic F127 solution was kept at 2 °C and was loaded into a syringe before print. PCL was dissolved in a mixture of DCM and DMF (60/30; *v*/*v*) at a total concentration of 10 wt % for electrospinning in 50% humidity. A voltage of 15 kV was applied across the setup during in the electrospinning process and the rate of feed was controlled at 500 μL/min.

### 2.4. Construction of the Structure with Multiscale Channels

**Figure 2:**
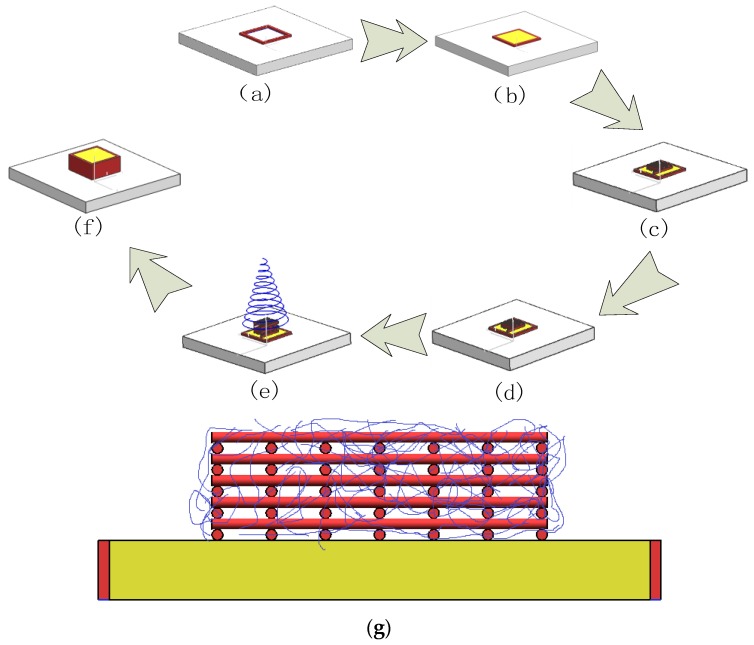
Process for the fabrication of constructs with multiscale channels: (**a**) patterned ring structure; (**b**) liquid gelatin solution poured into patterned ring structure to form base; (**c**) first layer of sacrificial material was printed on the base; (**d**) second layer of sacrificial material printed on first layer; (**e**) electrostatic spinning process after completing 6 layers; (**f**) complete ring structure and pouring of liquid gelatin solution into patterned ring structure; and (**g**) sectional view before encapsulation.

For bioprinting, Pluronic F127 and PCL were used as sacrificial materials to create multiscale channels in the gelatin matrix (Figure 2). Pluronic F127 hydrogel was printed on the platform using predesigned pattern. This step was repeated 3–5 times to pattern a ring structure (Figure 2a). Next, mTG powder was added to the 18% liquid gelatin solution and stirred rapidly to obtain the mTG/gelatin mixed solution. This mixed solution was poured into the patterned ring structure as the base of matrix. The next steps were carried out once the gelatin solidified (Figure 2b). According to the designed network structure, Pluronic F127 was printed on the base of the matrix to complete the first layer. To keep the printability of Pluronic F127, the environmental temperature was maintained at 25 °C (Figure 2c). Pluronic F127 was printed on the first layer to pattern the designed network structure (Figure 2d). The electrostatic spinning process was started after completion of 6 layers. PCL fibers were deposited on the 3D-printed macro-network structure forming the microchannel network structure. PCL electrostatic spinning occurs a rapid rate, and the required network structure was deposited in a short time of 30 s (Figure 2e). After electrostatic spinning was completed, several layers of Pluronic F127 were printed on the ring structure. This was followed by pouring liquid gelatin solution into the ring structure to encapsulate macro- and micro-network structures (Figure 2f). The sectional view before encapsulation can be seen, and the red represents F127 and the blue represents PCL (Figure 2g). The size of the construct is 10 × 10 × 5 mm^3^, and the macrochannels’ diameter was controlled at about 500 μm. Seven macrochannels were parallelly arranged in one layer, and then 10 layers were printed in construct. The entire structure was placed at 4 °C for 20–30 min to liquefy Pluronic F127 and to induce gelatin crosslinking. Based on our early experiments, the speed of gelatin solidification is faster than the speed of F127 liquefaction. So, before F127 had liquefied in 4 °C, the gelatin matrix has already solidified. The 3D matrix with macrochannels was incubated at 37 °C for 6 h to fully crosslink the hydrogel. Then, the entire construct with 3D network macrochannels was immersed into dichloromethane solution, then placed on a shaker (frequency of 100 r/min) inside an incubator to remove PCL for patterning the micro-network structure. Ethanol (75%) and dissolved dichloromethane were used for sterilization. Ultra Violet (UV) lamp irradiation was used for further sterilizing the construct. After completing all processes, the macrochannel surface in construct was seeded with HUVECs. This construct was kept in a culture medium for 7 days. Constructs without micro-networks and with PCL microfibers were prepared as controls. Three groups of constructs were fabricated: (a) with multiscale channel, which included both macrochannel and microchannel (Gel-F127-PCL) (b) with macrochannel and without microchannel (Gel-F127), and (c) without macrochannel and with PCL network, which have not been removed (Gel-PCL).

### 2.5. Construct Degradation

The gelatin construct was kept in a centrifuge tube immersed in 10 mL of phosphate-buffered saline (PBS), pH 7.4. This centrifuge tube was kept in a constant temperature (37 °C) vapor bath vibrator and oscillated at 60 r/min. Moreover, PBS in the centrifuge was replaced every week. The construct was cleaned with deionized water. Freeze-dried samples were weighted and analyzed after 6 h and 1 day, 3 days, 7 days, 14 days. Every group had 3 samples. Degradation rate of construct was calculated as:

*W*% = (*W*_1_ − *W*_2_)/W_1_ × 100%

where, *W*_1_ and *W*_2_ are weights of the primary and degraded construct, respectively.

### 2.6. Morphology

At the end of the preparation, the completed construct was observed using a scanning electron microscope (SEM) (SU1510, Hitachi, Japan). Five samples were prepared in every group. Two samples were cut along the section of sacrificial spinning, and three samples were cut into 5 × 5 × 5 mm^3^ cubes. All samples were dried using a vacuum freezing drying oven (Four-Ring Science Instrument Plant Beijing Co., Ltd., Beijing, China), and a conductive layer of gold was sputtered on the surface to avoid charging effects.

### 2.7. Viability Analysis

Five samples were prepared in every group for viability analysis. All samples were placed into a 70 °C water bath for 30 min to inactivate the residual mTG before the cell experiment. After that, the samples were immersed in ethanol (75%) for 12 h under UV radiation, and then the channels in the construct were washed with PBS three times. Finally, the construct was immersed in fresh RPMI 1640 medium for 6 h before injection. After trypsinization, a cell suspension with a density of 3 × 10^6^ cells/mL was prepared in RPMI 1640 medium. Then, 10 mL of cell suspension was slowly perfused into the macrochannels in the constructs by syringe (groups Gel-F127-PCL and Gel-F127). After 4 h of cell attachment, the cell-laden constructs were statically cultured, with the medium changed every day in a 5% CO_2_ incubator. Live-Dead Cell Staining Kit (BioVision, Inc., San Francisco, CA, USA) was used to assess cell viability and distribution within the construct. This kit utilizes a cell-permeable green fluorescent dye, Live-Dye, to stain live cells. Dead cells can be easily stained using propidium iodide (PI), a cell nonpermeable red fluorescent dye. Staining Solution for the construct was prepared using a mixture of 1 μL of Live-Dye and 1 μL of PI in 1 mL of Staining Buffer. The cell-laden construct with staining solution was incubated at 37 °C for 15 min, and then solutions were removed. The construct was washed three times with sterilized PBS and observed under an Olympus FluoView™ FV1000 laser scanning confocal microscope (Olympus NDT Inc., Boston, MA, USA) using the red and green fluorescence filters on days 1, 3, and 7. Three slides were used for the confocal microscope. ImageJ was used for the automated counting of red- and green-stained HUVECs in *z*-axis projections, and viable cell percentage was calculated.

### 2.8. Endothelialization Analysis

Five samples (group Gel-F127-PCL and group Gel-F127) were prepared for endothelialization analysis. All samples were placed into a 70 °C water bath for 30 min to inactivate the residual mTG before the cell experiment. After that, the samples were immersed in ethanol (75%) for 12 h under UV radiation, and then the channels in the construct were washed with PBS three times. Finally, the construct was placed in fresh RPMI 1640 medium for 6 h before injection. After trypsinization, a cell suspension with a density of 3 × 10^6^ cells/mL was prepared in RPMI 1640 medium. Then, 10 mL of cell suspension was slowly perfused into the macrochannels in the constructs. After 4 h of cell attachment, the cellular constructs were placed in fresh culture medium and statically cultured, with medium changed each day. The adhesion of HUVECs could be observed with a fluorescent microscope (Eclipse Ti-U, Nikon Instruments Inc., Tokyo, Japan). As for the endothelialization of HUVECs, tetramethylrhodamine (TRITC)-phalloidin and 4′,6-diamidino-2-phenylindole (DAPI) solutions (YEASEN) were used to stain cellular skeleton and nucleus. The cell–hydrogel constructs were fixed in 4% methanol solution and treated with 0.5% Triton X-100 solution. TRITC-phalloidin and DAPI solutions were subsequently injected into the macrochannel to stain endothelial cells on day 7. The cellular morphology and nucleus were observed with the fluorescent microscope.

## 3. Results

### 3.1. Construct Degradation Rate

**Figure 3:**
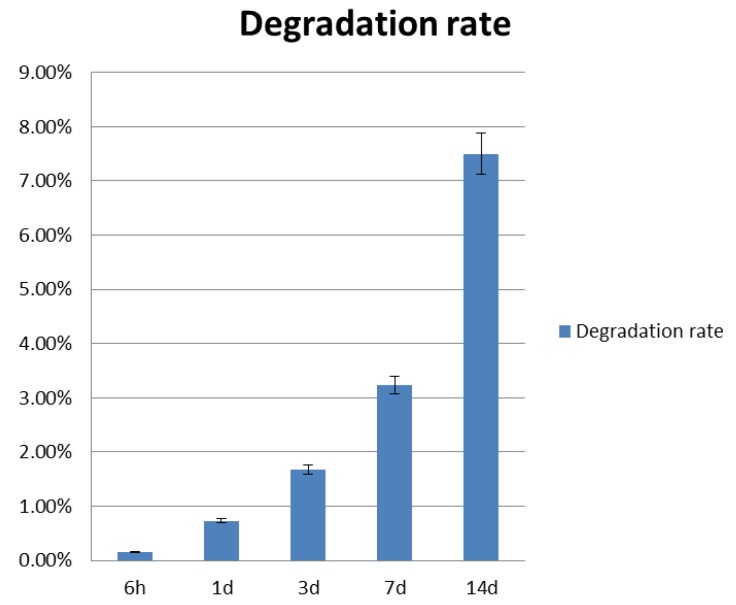
Degradation rate of the fabricated constructs.

Each group Gel-F127-PCL sample was weighed over fixed time intervals. Average weights were determined for each group. Degradation rates of the construct at 6 h and 1 day, 3 days, 7 days, and 14 days were 0.15%, 0.73%, 1.68%, 3.24%, and 7.5%, respectively (Figure 3). The degradation rate of the construct after 14 days is not considered high. Also, the trend in degradation rate can be considered low. The calcium alginate scaffold was fully degraded in PBS. After one week, the scaffold had disappeared in PBS.

### 3.2. Morphology and Channel Size

**Figure 4:**
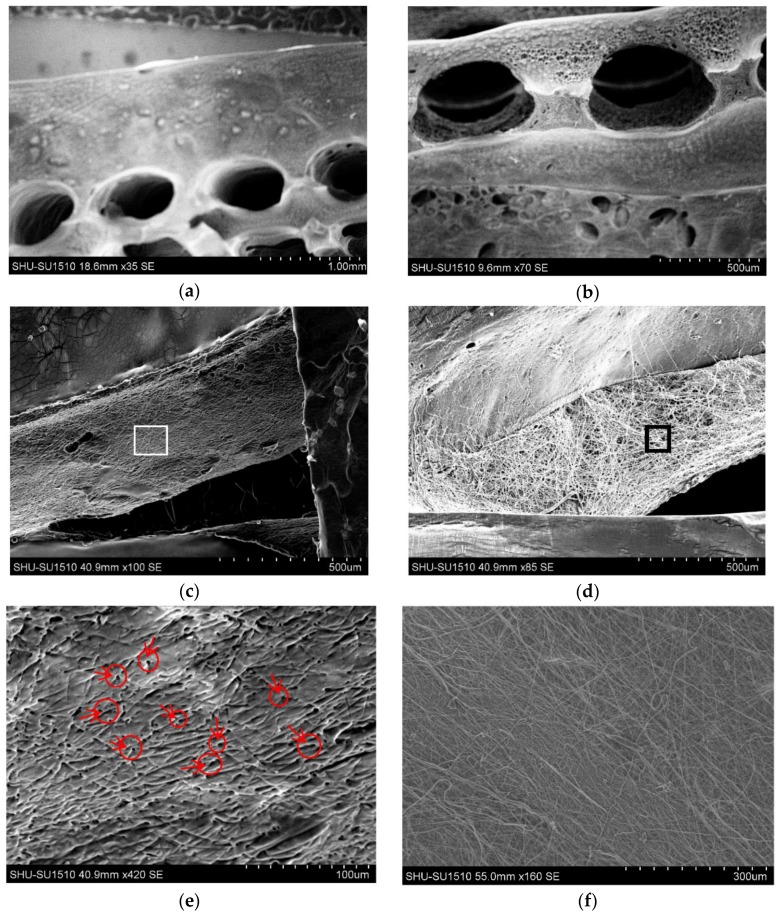
Scanning electron microscope images of the construction. (**a**,**b**) Orderly macrochannels in the construct; (**c**) section image of Gel-F127-PCL group; (**d**) section image of Gel-PCL group; (**e**) image of partial enlargement of (**c**); and (**f**) image of partial enlargement of (**d**). Red arrows and circles in (**e**) mark some small holes.

SEM images of the construct are shown in Figure 4. Three groups of construct were made using different processes. Both macro- and microchannels were fabricated, thus, multiscale channels were obtained in the gelatin matrix. Figure 4a,b show ordered macrochannels in the construct, and channel shape was kept intact after removal of F127. All construct groups showed macrochannels with channel sizes ranging from 400 to 600 μm. Figure 4c,d show a comparison between Gel-F127-PCL and Gel-PCL groups. The section of Gel-PCL group had many PCL micro-fibrous channels, as shown, and the section of the Gel-F127-PCL group had an obvious network structure. The Gel-F127-PCL showed microchannels with channel sizes ranging from 2 to 20 μm. Figure 4e shows a higher magnification of a region from Figure 4c. Figure 4e shows many small holes and small grooves, and it can be observed from the figure that the microchannel was formed after the sacrificial PCL was removed. Red arrows and circles have been added to the figure to mark some small holes. Figure 4f shows a higher magnification of a region from Figure 4d, and it shows many PCL fibers on the surface.

### 3.3. Channel Connectivity

**Figure 5:**
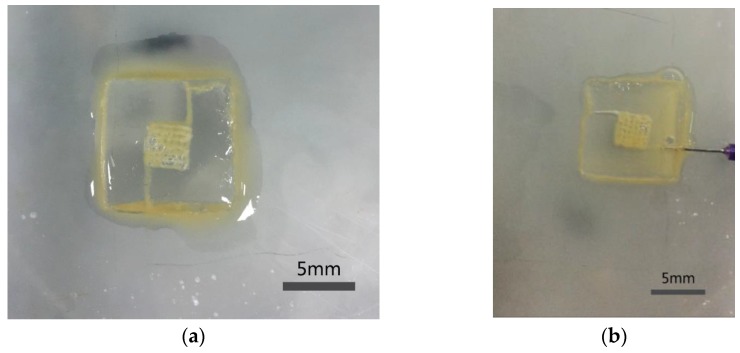
The connectivity experiment of construct. (**a**) The connectivity of macrochannels (**b**) the connectivity of multiscale channels. Scale bar is 5 mm.

The connectivity of the engineered structures was investigated using a syringe filled with yellow pigment. Figure 5 shows an image with the yellow pigment injected into the channels, which illustrates the connectivity of the channels. Figure 5a shows connectivity of the macrochannels in the construct (group Gel-F127). All macrochannels were filled with yellow pigment. Figure 5b shows connectivity of multiscale channels in the construct (group Gel-F127-PCL). When the yellow pigment was injected into the construct with multiscale channels, the entire construct had a yellow appearance, and all macrochannels and microchannels were filled. Connectivity of the channels tested here is critical, as it plays a significant role for vascularization to help cell metabolism. Scale bar in Figure 5 is 5 mm.

### 3.4. Viability Analysis of Seeded Human Umbilical Vein Endothelial Cells (HUVEC)

**Figure 6:**
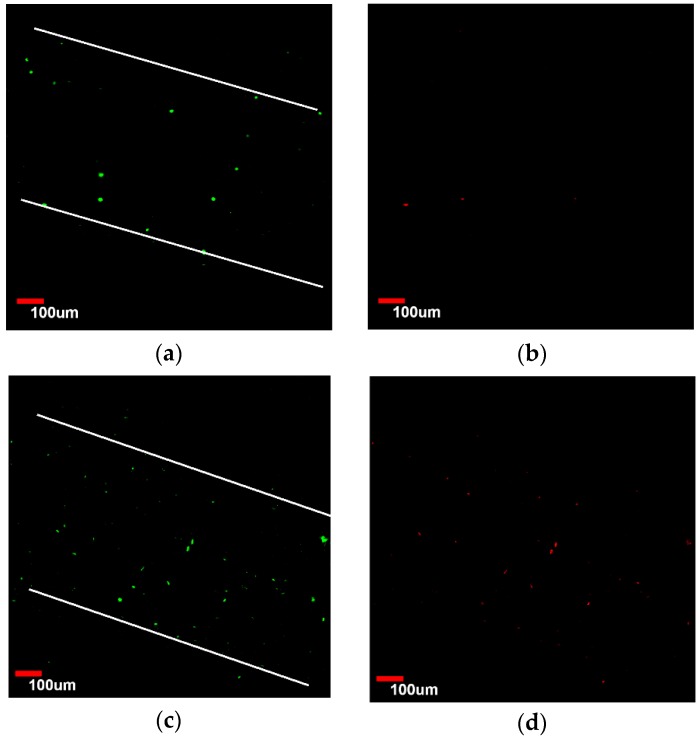

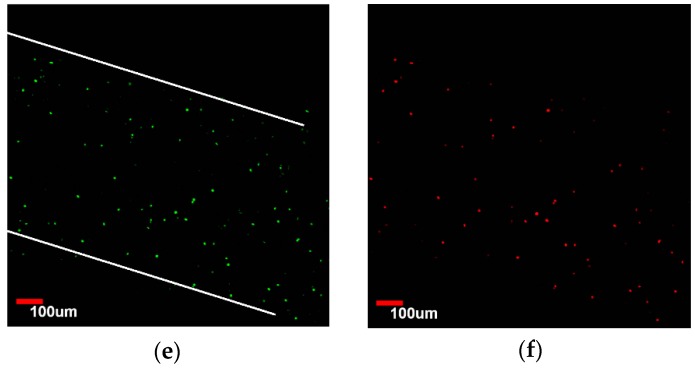
Laser confocal imaging of viability staining. Human umbilical vein endothelial cells (HUVECs) of group Gel-F127 labeled with Live-Dye and propidium iodide after seeding in the macrochannels. Images were taken using a confocal laser scanning microscope. Live and dead cells are fluorescent green and red, respectively. (**a**,**b**) About 17% dead cells were observed on day 1; (**c**,**d**) about 27% dead cells were visible on day 3; and (**e**,**f**) about 35% dead cells were obvious on day 7.

**Figure 7:**
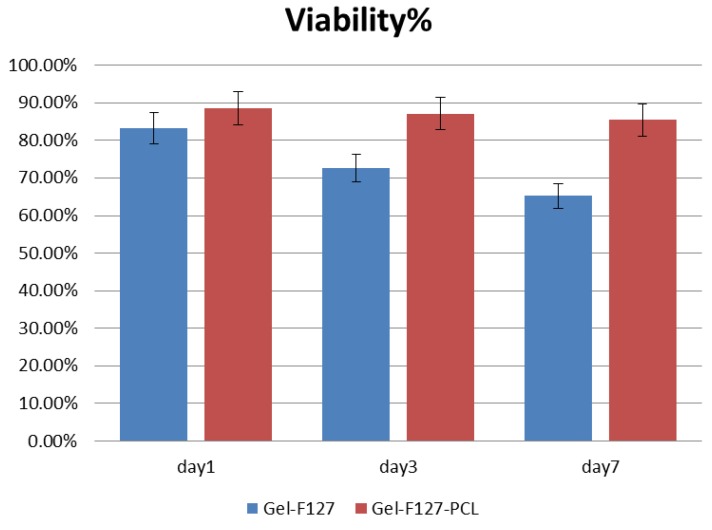
Time course of HUVECs viability after being seeded on group Gel-F127 and group Gel-F127-PCL. Cell viability was analyzed using ImageJ. The error bars indicate standard deviations.

After printing of the construct, cells were evenly seeded in the macrochannels. The cells were found to adhere to the channel surfaces after the fabrication process. After cells were seeded, viability of the HUVECs seeded on the surface of the channels was evaluated as a function of time. Figure 6a,b show the confocal microscopic image of viable and dead HUVECs of group Gel-F127 on day 1. Observation of the live HUVECs stained with green confirmed the distribution of cells in the macrochannels. Due to the limited magnification, only one macrochannel was observed. The white line in Figure 6 was added to label the observed channel structure. On day 3, the number of HUVECs in the macrochannel increased. Although most cells were found to be viable, some cells were stained with propidium iodide (red) (Figure 6c,d). After 7 days, a varied distribution of HUVECs was observed, and dead cells were visible (Figure 6e,f). ImageJ analysis revealed that initial cell viability was around 83.3% ± 4.21% on day 1 for cells seeded in the macrochannels. Cell viability decreased on day 3 (72.6% ± 4.53%) and day 7 (65.2% ± 3.87%) (Figure 7).

**Figure 8:**
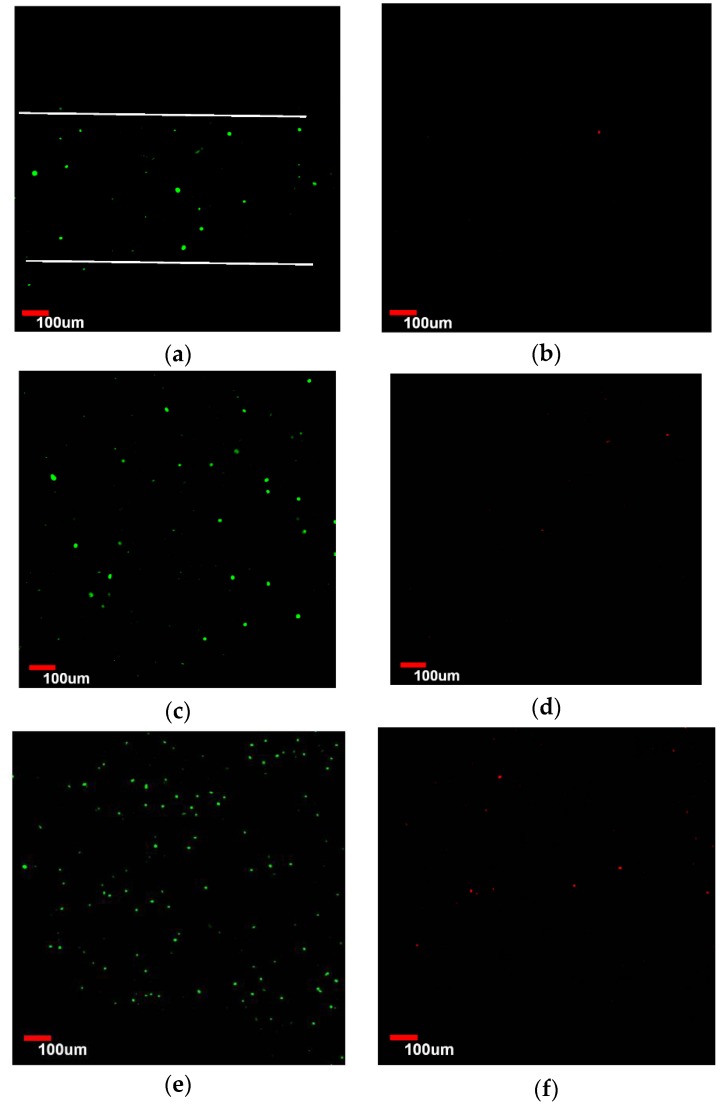
Laser confocal imaging of viability staining. HUVECs of the Gel-F127-PCL group labeled with Live-Dye and propidium iodide after seeding in the macrochannels in the construct with multiscale channels and imaged with confocal laser scanning microscope. Live and dead cells are fluorescent green and red, respectively. (**a**,**b**) About 10% dead cells were observed on day 1; (**c**,**d**) about 12% dead cells were visible on day 3; and (**e**,**f**) about 15% dead cells were obvious on day 7.

Figure 8a,b show confocal microscopic images of viable and dead HUVECs of the Gel-F127-PCL group at day 1. Observation of the live HUVECs stained with green confirmed a distribution of cells in the macrochannels in the Gel-F127-PCL group. On day 3, the number of HUVECs in the macrochannel increased, accompanied by cells showing the tendency to migrate in all directions. Similar to the previous case, a few cells were found to be stained with propidium iodide (red); however, most of the cells were still viable (Figure 8c,d). After 7 days, the distribution of HUVECs were strewn all around—just about 11% dead cells were visible (Figure 8e,f). ImageJ analysis revealed that initial cell viability was around 88.5% ± 5.41% on day 1 for cells seeded on the macrochannels, with cell viability being maintained on day 3 (87.1% ± 4.72% ) and day 7 (85.44% ± 4.96%) (Figure 7).

Results demonstrated that the engineered construct with macrochannels and multiscale channels had different cell viabilities. As seen from the results above, constructs with multiscale channels showed higher cell viability compared to the construct with macrochannels. Growth behavior of cells was also found to be different based on comparison of the laser confocal images. Cells in the construct with multiscale channels were observed to have a more prominent dispersion after 7 days. Moreover, cells in the construct without microchannels were closed to the construct with macrochannels.

### 3.5. Endothelialization Analysis of Seeded HUVEC

**Figure 9:**
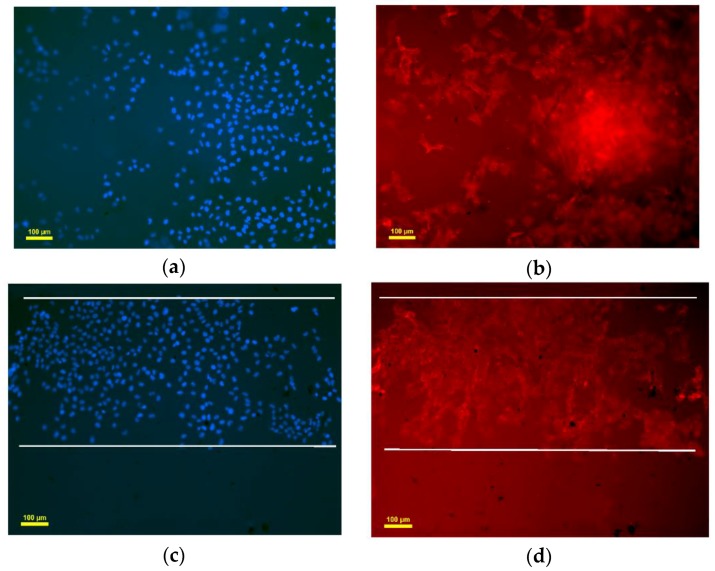
Group Gel-F127-PCL: (**a**) cell nucleus in the construct; (**b**) cytoskeleton in the construct; group Gel-F127: (**c**) cell nucleus in the construct; (**d**) cytoskeleton in the construct.

**Figure 10:**
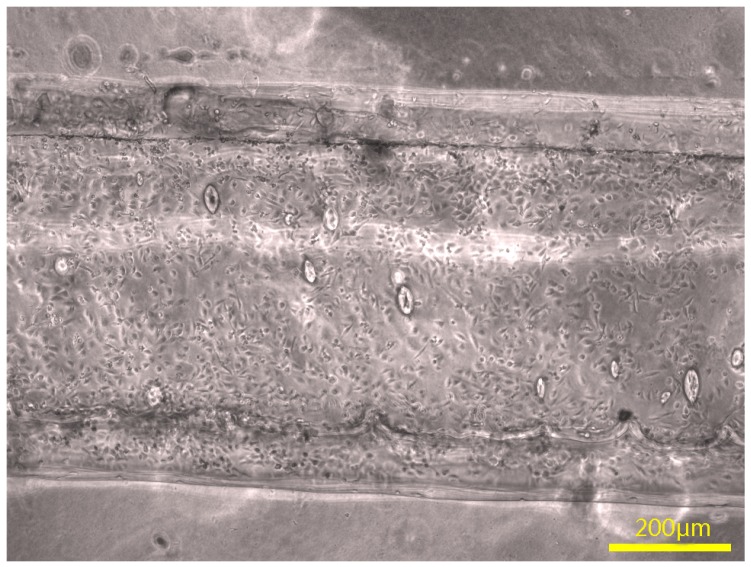
Adhesion of cells in the macrochannel.

After cells were seeded, endothelialization of the HUVECs seeded on group Gel-F127-PCL and group Gel-F127 was evaluated after 7 days. Figure 9a,b show the cells were diffused, which demonstrated the microchannels had worked. The blue dots represent cell nucleus, and the red fibers represent cytoskeleton. Cytoskeleton had connected, which demonstrated that the construct with multichannels was favorable to cell endothelialization. Figure 9c,d show macrochannels in the construct. The boundary of macrochannel has been marked with white line. No cells were observed between two macrochannels in group Gel-F127. Figure 10 shows HUVECs had adhered to the inner surface of the macrochannel. Cells were flat in the channels, which illustrated the adhesion of cells. Cells almost fully occupied the macrochannel on day 7.

## 4. Discussion

Recent research showed that bioprinting technologies had a revolutionary impact on the field of tissue engineering, organ manufacturing, and biological analysis [21]. Over the recent years, an increasing trend in research focus has been towards solving problems related to vascularization, which has been primarily achieved through structured constructs with embedded channels. In this study, longer electrostatic spinning time resulted in enough fibers followed by the formation of a thick film. This resulted in the separation of the construct along the film after the process to remove the PCL film was carried out. Thus, it can be concluded that electrostatic spinning time is an important process parameter that needs to be controlled precisely. A major limitation associated with biomaterials, which has been reiterated in research and reported in literature, is related to batch variation of biomaterials [22]. To alleviate these issues, many factors were taken into consideration before selecting materials in our study. For example, various parameters were taken into account before selecting the crosslinking agent (i.e., cost, cytotoxicity, crosslinking time, etc.). Cost of mTG was significantly lower in comparison to other crosslinking agents such as genipin. In terms of cytotoxicity, mTG has lower cytotoxicity compared to glutaraldehyde [23]. Moreover, mTG has a short crosslinking time (*t* = 10 min), which means short preparation time for the entire process.

In this work, we investigated a new method for engineering constructs with multiscale channels, using the inspiration from the blood vessel structure in the human body. We demonstrated feasibility via the above-discussed detection methods. Nowadays, the scale of channels in many researches have been single, and we have improved the current situation. The scaffold with channels could help to solve the problem about vascularization, so we hope the construct with multiscale channels can achieve a better effect. The highest cell viability in the construct with multiscale channels could maintain above 90%, and we could observe diffusion of cells, which demonstrated the function of microchannels. We perform more biological detection methods in the future, including experiments in mice to verify biocompatibility, with our final goal being application of this construct to the repair of damaged tissues. Now, many improvements can be obtained in this construct. We will utilize electrostatic spinning layer-by-layer in the next experiment, which results in microchannels in the construct. Controllable electrostatic spinning on the construct is also a research issue, because concentration of capillaries in the body varies. We will also seed other cells on this construct, such as smooth muscle cells and fibroblasts to simulate vascularization scaffolding, and culture cells for longer periods of time. Moreover, we will add growth factor to the construct to promote vessel growth using dynamic cultures in the future.

## 5. Conclusions

In this research, a novel method for the biofabrication of engineered constructs with multiscale channels was presented. Such a design shows great potential due to improved vascularization. As a result, this method can be used to fabricate complex tissues of living organs in the near future.

Results showed that the fabricated construct with multiscale channels is a promising method and can be used for enhancing vascularization in tissue-engineered regeneration with the following advantages: (1) construction with multiscale channels can be achieved easily using bioprinting and sacrificial technology; (2) materials used in this method were biodegradable, biocompatible, with no cytotoxicity and no byproducts produced after crosslinking; (3) arbitrary shape of constructs with multiscale channels can be produced by bioprinting (i.e., there are no restrictions related to shape and design).

Here, the construct with multiscale channels fabricated using bioprinting and sacrificial technology was used to generate the structure. Macrochannels and microchannels in the construct were observed to show excellent connectivity. Moreover, the degradation rate was not fast, which could keep the HUVECs growing on the scaffold to allow vascularization. Results also showed that there was no internal blockage in the engineered construct. Application of HUVECs showed high cell viability and a diffusion phenomenon in the construct with multiscale channels. Thus, this method can be used as a practical platform for the fabrication of engineered construct for vascularization.

## References

[1] Puelacher W.C., Vacanti J.P., Ferraro N.F., Schloo B., Vacanti C.A. (1996). Femoral shaft reconstruction using tissue-engineered growth of bone. Int. J. Oral Maxillofac. Surg..

[2] Tian L., George S.C. (2011). Biomaterials to prevascularize engineered tissues. J. Cardiovasc. Transl. Res..

[3] Jain R.K., Au P., Tam J., Duda D.G., Fukumura D. (2005). Engineering vascularized tissue. Nat. Biotechnol..

[4] Ling Y., Rubin J., Deng Y., Huang C., Demirci U., Karp J.M., Khademhosseini A. (2007). A cell-laden microfluidic hydrogel. Lab Chip.

[5] Sakaguchi K., Shimizu T., Horaguchi S., Sekine H., Yamato M., Umezu M., Okano T. (2013). In vitro engineering of vascularized tissue surrogates. Sci. Rep..

[6] Xu C., Zhang Z., Christensen K., Huang Y., Fu J., Markwald R.R. (2014). Freeform Vertical and Horizontal Fabrication of Alginate-Based Vascular-Like Tubular Constructs Using Inkjetting. Manuf. Sci. Eng..

[7] Hammer J., Han L.H., Tong X., Yang F. (2014). A facile method to fabricate hydrogels with microchannel-like porosity for tissue engineering. Tissue Eng. Part C Methods.

[8] Yoo S.M., Ghosh R. (2014). Fabrication of alginate fibers using a micro-porous membrane based molding technique. Biochem. Eng. J..

[9] Zhang Y., Yu Y., Chen H., Ozbolat I.T. (2013). Characterization of printable cellular micro-fluidic channels for tissue engineering. Biofabrication.

[10] Lee V.K., Kim D.Y., Ngo H., Lee Y., Seo L., Yoo S.S., Vincent P.A., Dai G.H. (2014). Creating perfused functional vascular channels using 3D bio-printing technology. Biomaterials.

[11] Kolesky D.B., Truby R.L., Gladman A.S., Busbee T.A., Homan K.A., Lewis J.A. (2014). 3D Bioprinting of Vascularized, Heterogeneous Cell-Laden Tissue Constructs. Adv. Mater..

[12] Guillemot F., Mironov V., Nakamura M. (2010). Bioprinting is Coming of Age: Report from the International Conference on Bioprinting and Biofabrication in Bordeaux. Biofabrication.

[13] Mironov V., Reis N., Derby B. (2006). Bioprinting: A beginning. Tissue Eng..

[14] Calvert P. (2001). Inkjet Printing for Materials and Devices. Chem. Mater..

[15] Ozbolat I., Yu Y. (2013). Bioprinting toward Organ Fabrication: Challenges and Future Trends. IEEE Trans. Biomed. Eng..

[16] Mironov V. (2003). Printing Technology to Produce Living Tissue. Expert Opin. Biol. Ther..

[17] Gandhimathi C., Venugopal J.R., Tham A.Y., Ramakrishnam S., Kumar S.D. (2014). Biomimetic hybrid nanofibrous substrates for mesenchymal stem cells differentiation into osteogenic cells. Mater. Sci. Eng. C.

[18] Shamaz B.H., Anitha A., Vijayamohan M., Kuttappan S., Nair S., Nair M.B. (2015). Relevance of fiber integrated gelatin-nanohydroxyapatite composite scaffold for bone tissue regeneration. Nanotechnology.

[19] Johnson H., Sina N., Zhilian Y., Robert K., Anita Q., Simon E., Gordon G. (2013). Bio-Ink Properties and Printability for Extrusion Printing Living Cells. Biomater. Sci..

[20] Baier Leach J., Bivens K.A., Patrick C.W., Schmidt C.E. (2003). Photocrosslinked hyaluronic acid hydrogels: Natural, biodegradable tissue engineering scaffolds. Biotechnol. Bioeng..

[21] Figeys D., Pinto D. (2000). Lab-on-a-chip: A revolution in biological and medical sciences. Anal. Chem..

[22] Zhao Y., Li Y., Mao S., Sun W., Yao R. (2015). The influence of printing parameters on cell survival rate and printability in microextrusion-based 3D cell printing technology. Biofabrication.

[23] He J., Chen R., Lu Y., Zhan L., Liu Y., Li D., Jin Z. (2016). Fabrication of circular microfluidic network in enzymatically-crosslinked gelatin hydrogel. Mater. Sci. Eng. C.

